# Bilateral Schwartze Sign, Decision-Making for Surgery

**Published:** 2013-10

**Authors:** Morteza Nourollahian, Shirin Irani

**Affiliations:** 1 *Department of Otorhinolaryngology, Imam Reza Hospital. Faculty of medicine, Mashhad University of Medical Sciences*; 2 *Department of Otorhinolaryngology, Mashhad University of Medical Sciences*

Otosclerosis is an otic capsule disorder that leads to progressive conductive and/or sensorineural hearing loss due to stapes footplate fixation and cochlear bone involvement.

The disorder is bilateral in 70% of patients and it usually starts between the third and fifth decade. One of its characteristic clinical findings is Schwartze sign which refers to a reddish discoloration over the promontory seen beyond the intact tympanic membrane. Schwartze sign, also known as Flemingo's flush sign or Rising sun sign is believed to be associated with otospongiosis which is the active phase of the disease.

The prevalence of Schwartze sign is estimated to be 10%. The prevalence of bilateral sign has not been reported, but it is presumed to be too low.

**Fig 1:**
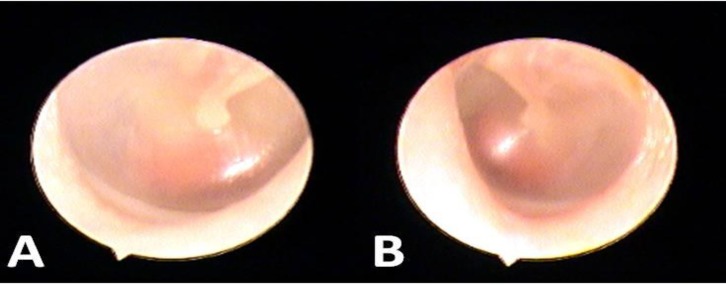
Ootoscopic View

Herein, we introduce a patient with bilateral positive Schwartze sign. A twenty two year old woman with bilateral hearing loss came to our clinic. She also complained from non pulsating tinnitus in both ears since one year ago. On physical examination, bilateral schwartze sign with an intact tympanic membrane was noted (Fig.1).

The audiometric evaluation showed a conductive hearing loss with speech reception threshold of 50 dB and 30 dB for the right and left ear respectively. Sodium fluoride 8mg every 8 hours was prescribed for three months. She noticed partial improvement during the treatment and on her otoscopic exam, the Schwartze sign was reduced. Then stapedotomy was scheduled for the right ear (the worse ear in regard to the hearing & tinnitus). The surgery went uneventful and she proved to be much better afterwards. Her right ear tinnistus has gone completely and the speech reception threshold has changed from 45 dB preoperatively to 10 dB postoperatively. 

It seems that the surgery remains a therapeutic option, when the active phase of the disease is stabilized even following a short course of pharmacologic therapy. 

